# The efficiency and safety of steroid addition to multimodal cocktail periarticular injection in knee joint arthroplasty: a meta-analysis of randomized controlled trials

**DOI:** 10.1038/s41598-019-43540-9

**Published:** 2019-05-07

**Authors:** Zhenhan Deng, Yusheng Li, Garrett R. Storm, Ronak Naveenchandra Kotian, Xuying Sun, Guanghua Lei, Shanshan Gao, Wei Lu

**Affiliations:** 1 Department of Sports Medicine, the First Affiliated Hospital of Shenzhen University, Shenzhen Second People’s Hospital, Shenzhen, Guangdong China; 2 Department of Orthopaedics, Xiangya Hospital, Central South University, Changsha, Hunan China; 3 National Clinical Research Center for Geriatric Disorders, Xiangya Hospital, Central South University, Changsha, Hunan China; 4 Department of Cardiology, University of Colorado Denver, Aurora, Colorado USA; 5 Department of Orthopaedics, Victoria Hospital, Bangalore Medical College and Research Institute, Bangalore, India; 6 Department of Orthopaedics, Biological Engineering and Regenerative Medicine Center, Tongji Hospital, Huazhong University of Science and Technology, Wuhan, Hubei China

**Keywords:** Osteoarthritis, Osteoarthritis

## Abstract

Steroids are frequently used for postoperative pain relief without definite evidence. This study was conducted to assess the pain management effect of the addition of steroids to a multimodal cocktail periarticular injection (MCPI) in patients undergoing knee arthroplasty and evaluate their safety. Pubmed, Embase, and Cochrane Library were searched through April, 2018. A total of 918 patients from ten randomized controlled trials (RCTs) were ultimately included. Compared with placebo groups, steroids application could effectively relieve pain on postoperative day (POD)1; decrease C-Reactive protein (CRP) level on POD3; improve range of motion (ROM) in postoperative 5 days; reduce morphine consumption, achieve earlier straight leg raising (SLR), and shorten the length of stay (LOS) in hospital. With regards to adverse effects, it did not increase the risk of postoperative infection, postoperative nausea and vomiting (PONV), or other complications. However, no significant difference in pain relief, ROM, or increased Knee Society Knee Function Scores were found during long-term follow up. Overall, this meta-analysis ensured the efficiency and safety of steroids with MCPI in knee arthroplasty patients during the early postoperative period.

## Introduction

Knee osteoarthritis (KOA) is a degenerative disease involving the intra-articular tibiofemoral and patellofemoral cartilage^1^. It causes chronic pain, functional limitation, and emotional disturbance which may lead to disability and poor quality of life^2^. A knee arthroplasty (KA) is a reliable and suitable surgical procedure for end-stage OA patients to relieve pain, to recover function and to improve health related quality of life^3^. With the creative designs and improved skills, we are now entering a new phase in which partial osteoarthritic changes can be treated with partial resurfacing prosthetic solutions such as unicompartmental, bi-unicompartmental or patellofemoral arthroplasty^4^. However, no matter what type of KA is performed, this surgery has commonly been associated with severe postoperative pain as well as postoperative nausea and vomiting (PONV). This can result in immense discomfort, emotional distress, low satisfaction, and further leads to poor surgical outcomes and delayed postoperative recovery^5,6^. Therefore, effective management of postoperative pain is essential for early rehabilitation and better functional outcomes. Various procedures including continuous epidural anesthesia, local infiltration anesthesia, peripheral nerve block, and patient controlled analgesia (PCA) are taken to control pain.

As an orthopedic surgeon, we place great concerns on the multimodal cocktail periarticular or intra-articular injection (MCPI) strategy after KA for analgesia. Not only this treatment is proven to be a safe and cost-effectiveness measure^7,8^, but also this intervention is easy to perform. Although the gold standard for the cocktail formula has not yet been set up, the components always contains local anesthetics (ropivacaine and bupivacaine), epinephrine, steroids, opioids and nonsteroidal anti-inflammatory drugs (NSAIDs)^9–11^. However, whether those compositions are all necessary is still to be investigated considering the efficacy and safety.

Steroids have been extensively used in various perioperative settings due to their potent anti-inflammatory and antiemetic effects^12,13^. Clinical trials have indicated that the addition of steroids to MCPI might decrease edema and blood loss and achieve permanently better range of motion (ROM) due to reduced local inflammatory response following surgical trauma^14,15^. Some studies have advocated the use of steroids for the benefits of postoperative pain relief and reduced risk of POVN^16–18^, while others have found no significant decrease in pain and PONV with administration of steroids^19,20^. Moreover, periarticular steroids use may arouse concerns for possible postoperative infection and patellar tendon rupture^21^. To sum up, it remains inconclusive whether the addition of steroids in the MCPI is necessary.

Several meta-analyses have been launched to address the efficacy and safety of steroids application in knee joint arthroplasty. The major drawback of these studies is not distinguishing local usage of steroid from systemic application^12,22–28^, which may hamper the reliability of the conclusion. Only three studies focused on the periarticular application of steroids in KA^26,27,29^. In Tran’s study, only five studies were included and there was no overwhelming data to suggest the addition of steroids to MPCPI improves postoperative total knee arthroplasty (TKA) pain^26^. In Zhao’s study, only six RCTs met the inclusion criteria and it was found that the addition of steroids to MCPI could improve the analgesic effect and was proved to be highly safe in patients undergoing TKA, but it couldn’t increase the postoperative ROM of knee joint^27^. Though eight studies were evaluated in Xing’s meta-analysis, it didn’t assess the analgesia effect of the steroid in patients underwent joint arthroplasty, the results showed that intraarticular steroid injections may lead to increased deep infection rates of subsequent joint arthroplasty^29^. Moreover, all of these studies were confined to TKA with a limited number of studies included. Actually, for the patients suffering KA, no matter whether partial knee arthroplasty (PKA) or TKA are performed, the mechanisms of pain, stress and other reactions that caused by KA might be similar^30–32^. Consequently, the aim of this study is to systematically summarize the available scientific literatures on all kinds of KA to evaluate the efficacy and safety the addition of steroids addition to MCPI by conducting a quantitative meta-analysis including randomized controlled trials (RCTs).

## Results

### Search results

**Figure 1:**
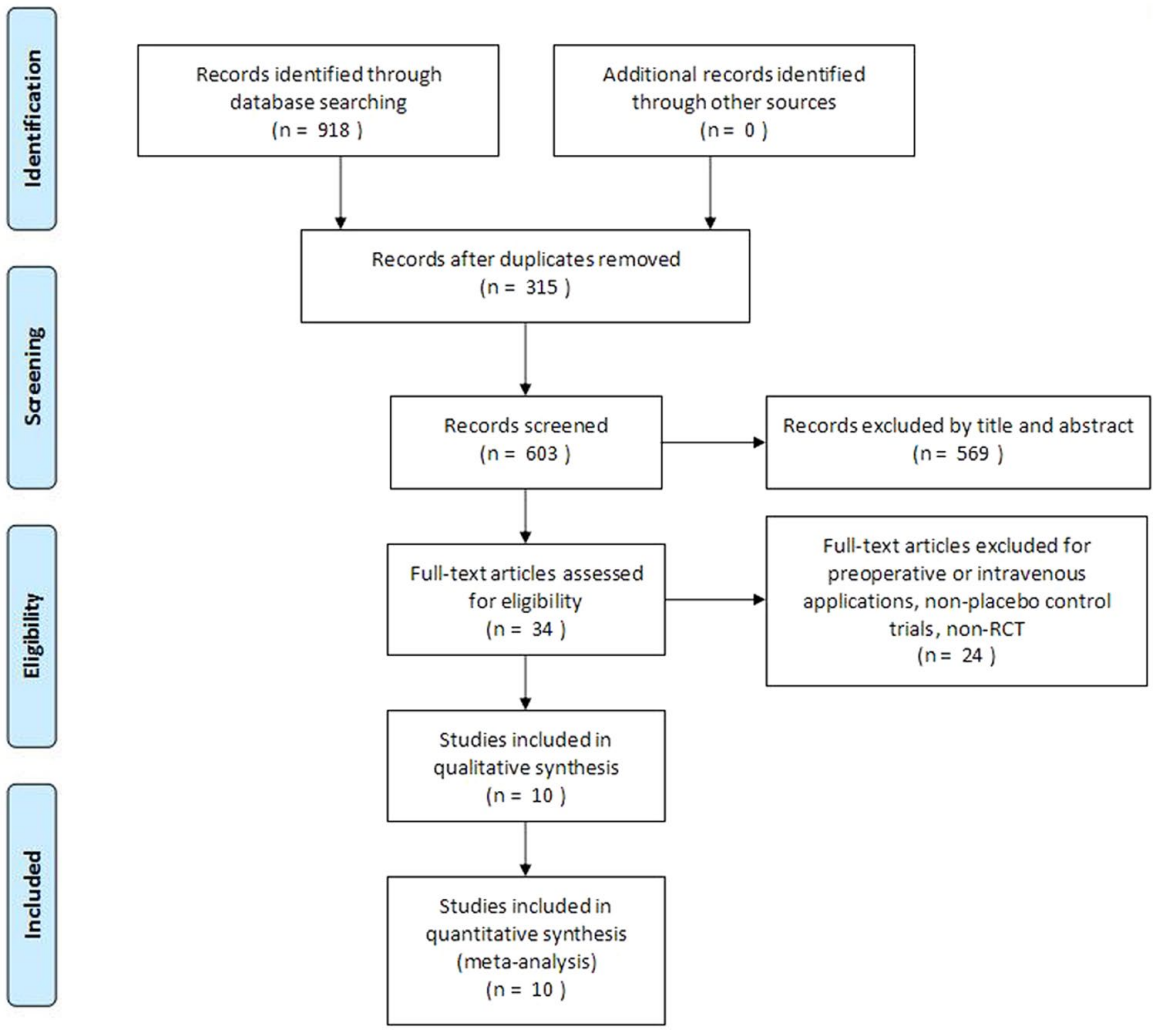
Flow chart of studyselection.

A total of 918 potentially relevant articles wereidentified from the databases, including 315 duplicated articles; 698 studies were excluded after screening titles and abstracts. 34 full-text articles were assessed for eligibility, of which 24 were excluded after assessment of the full-text articles. Eventually, ten^14,21,33–40^ articles that fulfilled the inclusion criteria were identified for synthetic evaluation. The flow diagram is presented in Fig. 1.

### Study characteristics

Demographic characteristics, the details about the included studies are summarized in Table 1. Ten RCTs were performed in 7 countries, involving 820 participants. Eight^21,33–39^ of the ten trials performed TKA surgery, while the other two^21,40^ performed UKA. Both experimental and control groups were tightly matched in sample size, age, sex, and body mass index (BMI). Experimental groups received single-dose postoperative intraarticular injection or periarticular infiltration of steroids, while control groups received placebo or none. Four studies^33–35,38^ performed spinal anesthesia (SA), two^36,37^ opted for general anesthesia (GA), one^21^ received endotracheal anaesthesia (EA), and the other three^14,39,40^

**Table 1:** Characteristics of ten included RCTs.

Study	Year of publication	Country	Surgery	Steroids group					No-Steroids group					Anaesthesia	Perioperative analgesia	Follow-up
				N	Mean age (years)	Sex (Male/female)	BMI (Kg/m^2^)	Intervention	N	Mean age (years)	Sex (Male/female)	BMI (Kg/m^2^)	Intervention			
Chia	2013	Australia	TKA	42	68.9 ± 8.0	NA	30.85 ± 5.5	TA 40 mg (+100 ml 0.2% ropivacaine +1:1000 adrenaline)	42	65.09 ± 8.4	NA	31.49 ± 4.7	No TA (+others)	SA	Postopertive oral celecoxib + oxycodone, dextropropoxypheneand paracetamol	12 weeks
Christian	2009	USA	TKA	39	65.8 ± 11.1	16/23	32.9 ± 6.5	MP 40 mg +bupivacaine 80 mg + morphine 4 mg +epinephrine 300 mg + clonidine 100 mg + cefuroxime 750 mg)	37	65.2 ± 11.0	7/30	35.1 ± 8.0	No MP (+others)	EA	Preoperative oral celecoxib + oxycodone hydrochloride/oxycodone and acetaminophen preoperatively	12 weeks
Ikeuchi	2014	Japan	TKA	20	77 ± 6	2/18	NA	DA 6.6 mg (+0.75%ropivacaine + isepamicin 400 mg)	20	76 ± 3	4/16	NA	No DA (+others)	GA	Postoperative PCA + oral loxoprofen + fentanyl injection	12 weeks
Kim	2015	Korea	TKA	43	71.4 ± 4.7	41/2	25.8 ± 3.3	MP 40 mg (+ropivacaine 180 mg + morphine 5 mg + ketorolac30 mg)	45	70.6 ± 5.5	39/4	27.2 ± 4.0	No MP (+others)	SA	Preoperative oral celecoxib + tramadol; Postoperative PCA + oral celecoxib + tramado + pethidineintramuscular injection (rescue) + oral oxycodone (rescue)	1 week
Kwon	2014	Korea	TKA	76	69.3 (62–77)	0/76	25.9 (22–32)	TA 40 mg (+morphine 10 mg + ropivacaine 300 mg + ketorolac 30 mg + 1:1000 of epinephrine300 ug)	76	69.3 (62–77)	0/76	25.9 (22–32)	No TA (+others)	SA	PostoperativePCA + oral celecoxib and ultracet + ketoprofenintramuscular injection (rescue)	6 months
Ng	2011	Singapore	UKA	41	63 (53–71)	10/31	28 (23–32)	TA 40 mg (+0.5% bupivacaine + 1:200,000 epinephrine)	42	62 (55–70)	11/31	27 (21–32)	No TA (+others)	SA or GA	Postoperative PCA + oral Synflex + oral amotidine	6 months
Pang	2008	Singapore	UKA	45	68 (54–80)	8/37	27.3 ± 6.1	TA 40 mg (+0.5 ml/kg of 1:200,000 epinephrine + 0.5% bupivacaine)	45	67 (44–80)	8/37	27.5 ± 5.6	No TA (+others)	SA or GA	Postoperative PCA + oral naproxen	2 years
Seah	2011	Singapore	TKA	29	67.9	NA	26.7	TA 40 mg (+0.5 ml/kg of 1:200,000 epinephrine + 0.5% bupivacaine)	30	65.4	NA	27.3	No TA (+others)	SA or GA	Postoperative PCA + oral naproxen	2 years
Tsukada	2016	Japan	TKA	38	75 (58–88)	5/35	26.7 (20.5–38.6)	MP 40 mg (+ropivacaine 300 mg + morphine 8 mg + epinephrine 0.3 mg + ketoprofen 50 mg	37	72 (47–88)	32/5	27.3 (18.4–40.6)	No MP (+others)	SA	Postoperative oral loxoprofen + diclofenacsodium (rescue)	1 year
Yue	2013	China	TKA	36	70.2 ± 6.4	32/4	25.23 ± 4.81	BA 1 mg (+30 ml 0.75% ropivacaine + 0.5 ml1:1000 adrenaline)	36	69.3 ± 5.7	32/4	26.14 ± 3.27	No BA (+others)	GA	Preoperative celecoxib; Postoperative PCA + oral celecoxib + morphine intramuscular injection (rescue)	1 year

Surgery: TKA, total knee arthroplasty; UKA, unicondylar knee arthroplasty.

Intervention: BA, betamethasone; DA, dexamethasone; MP, methylprednisolone; TA, triamcinoloneacetonide.

Anaesthesia: SA, spinal anaesthesia; EA, endotracheal anaesthesia; GA, general anaesthesia.

Perioperative analgesia: PCA, patient-controlled analgesia.

NA, not available.

received SA or GA. PCA with opioid, was given to all participates for concomitant pain management. The follow-up period ranged from 1 week to 2 years.

### Risk of bias assessment

Cochrane Collaboration tool was used to assess the RCTs (Table 2). Double blinding was provided in all RCTs. The randomization algorithm was mentioned in 6 trials^14,21,33,37–39^, including random number generator, block randomization, random numbers, and randomization table. Four trials^34–36,40^ recorded the allocation concealment information by sealed envelopes. Other assessments, such as blinding of participants and personnel, blinding of outcome assessment, incomplete outcome data, and selective reports, were all in good performance.

**Table 2:** Methodological quality assessment according to the Cochrane Collaboration’s Risk of Bias tool study.

Study	Random sequence generation	Allocation concealment	Blinding of participants and personnel	Blinding of outcome assessment	Incomplete outcome data	Selective reporting	Other bias
Chia	+ (a)	+*	+	+	+	+	?
Christian	+ (b)	?	+	+	+	+	?
Ikeuchi	+ (c)	+*	+	+	+	+	?
Kim	?	?	+	+	+	+	?
Kwon	?	?	+	+	+	+	?
Ng	?	?	+	+	+	?	?
Pang	+ (d)	?	+	+	+	+	?
Seah	+ (d)	+*	+	+	+	+	?
Tsukada	+ (c)	+*	+	+	+	+	?
Yue	?	?	+	+	?	+	?

+, low risk of bias; −, high risk of bias; ? unclear risk of bias.

Random sequencegeneration: a, random number generator; b, block randomization; c, random numbers; d, randomization table.

Allocationconcealment: *sealed envelopes.

### Visual analogue pain score (VAS)

**Figure 2:**
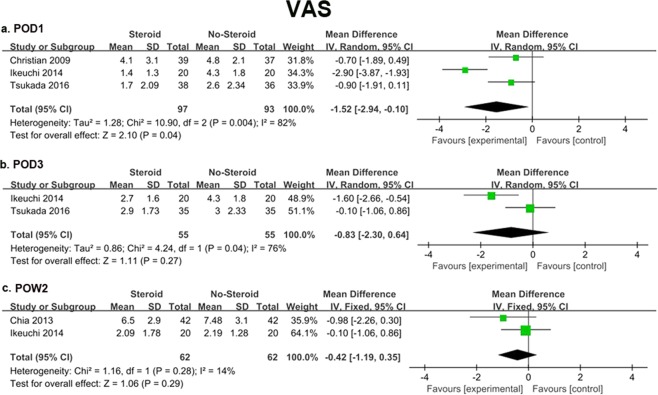
Forest plot diagram showing VAS at POD1 (**a**), POD3 (**b**), POW2 (**c**).

All studies recorded pain score, but only 4 trials^21,33,37,38^ provided detailed data as means and standard deviations (SDs). Two studies^33,38^ reported both VAS at rest (RVAS) and during activity (AVAS). AVAS could not be collected for analysis due to unsuitable format of the data. Therefore, if not specifically mentioned, VAS at different time points were all considered as RVAS. Finally, postoperative day (POD) 1, 3 and postoperative week (POW) 2 were eligible for meta-analysis. The result showed that VAS was significantly lower in the steroids group than the control group on POD1 (MD = −1.52, 95% CI −2.94 to −0.10, *P* = 0.04). However, no significant difference was found between the two groups on POD3 and POW 2. Random-effect models were used to perform meta-analysis for POD1 (*I*^2^ = 82%, *P* = 0.004) and POD3 (*I*^2^ = 76%, *P* = 0.04) as the substantial heterogeneity was high (Fig. 2).

### Range of motion (ROM)

**Figure 3:**
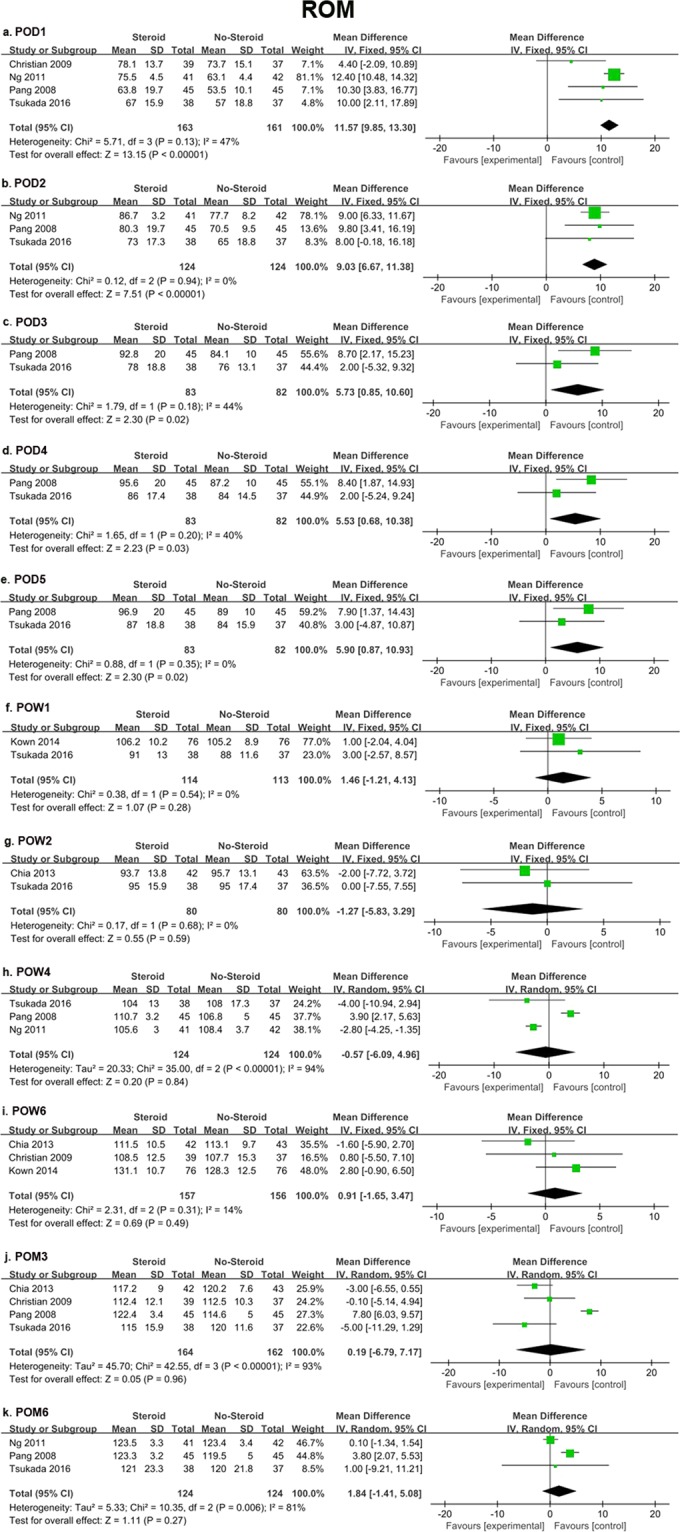
Forest plot diagram showing ROM at various time points after knee joint arthroplasty.

Seven studies^14,21,33,35,37,38,40^ provided detailed data. Eleven time points were analyzed which included POD1, 2, 3, 4, 5, POW1, 2, 4, 6, and postoperative month (POM) 3, 6, respectively. The results showed that the steroids group had significantly better ROM on the first postoperative 5 days: POD1 (MD = 11.57, 95% CI 9.85 to 13.30, *P* < 0.00001), POD2 (MD = 9.03, 95% CI 6.67 to 11.38, *P* < 0.00001), POD3 (MD = 5.73, 95% CI 0.85 to 10.60, *P* = 0.02), POD4 (MD = 5.53, 95% CI 0.68 to 10.38, *P* = 0.03), and POD5 (MD = 5.90, 95% CI 0.87 to 10.93, *P* = 0.02). However, the difference was not significant after POW1. The substantial heterogeneity was high at POW4 (*I*^2^ = 94%, *P* < 0.00001), POM3 (*I*^2^ = 93%, *P* < 0.00001), and POM6 (*I*^2^ = 81%, *P* = 0.006). Therefore, the random-effect models were used for these outcomes. Fixed-effect models were applied at other time points (Fig. 3).

### Morphine consumption

**Figure 4:**
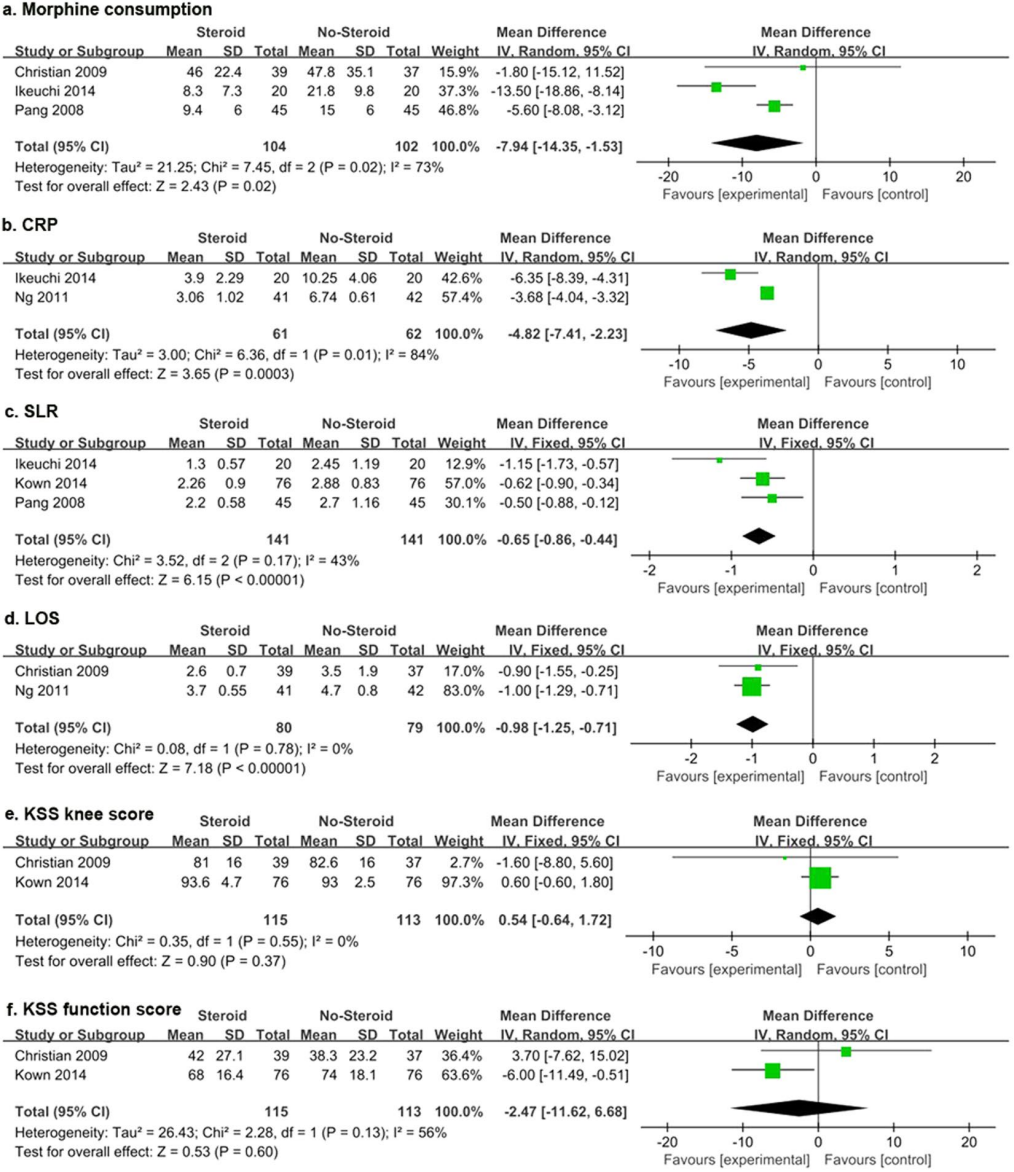
Forest plot diagram showing postoperative morphine consumption (**a**), CRP at POD3 (**b**), the interval to perform SLR postoperatively (**c**), LOS (**d**), KSS knee score (**e**) and function score (**f**) at POW6.

Three studies^14,21,37^ provided detailed data for postoperative morphine consumption and the total amount used during hospitalization was accumulated for analysis. A random-effect model was used due to significant heterogeneity (*I*^2^ = 68%, *P* = 0.02). The overall effect showed that morphine consumption for the steroid groups were significantly less than that for the control groups (MD = −7.94, 95% CI −14.35 to −1.53, *P* = 0.02; Fig. 4a).

### C-reactive protein (CRP)

Three studies^37,38,40^ provided detailed data for CRP level, but only one time point included two studies that were eligible for meta-analysis. Pooled results showed that the CRP level on POD3 in the steroid groups was significantly lower than that in control groups (WMD = −4.82, 95% CI −7.41 to −2.23, *P* = 0.0003). Random-effect models were used for this study (*I*^2^ = 84%, *P* = 0.01; Fig. 4b).

### Straight leg raising (SLR)

The interval required to perform a SLR was collected from three studies^14,35,37^ involving 282 knees. The result indicated that the patients in the steroid groups could perform a SLR significantly earlier than those in the control groups (MD = −0.65, 95% CI −0.86 to −0.44, *P* < 0.00001). Statistical heterogeneity was not found in SLR (*I*^2^ = 43%, *P* = 0.17). A fixed-effects model was performed (Fig. 4c).

### Length of stay (LOS)

Two studies^21,40^ provided detailed data with regard to length of stay in hospital. The results indicated that the patients in the steroid groups had significantly shorter LOS than that of the control groups (MD = −0.98, 95% CI −1.25 to −0.71, *P* < 0.00001). The fixed-effects model was selected as no significant heterogeneity was found (*I*^2^ = 0%, *P* = 0.78; Fig. 4d).

### Knee society knee and function scores (KSS)

Two studies^21,35^ provided detailed data of KSS knee scores and function scores, respectively. Only 1 time point was eligible for meta-analysis. During the follow-up evaluation POM6, the patients in the steroid groups did not achieve higher KSS knee scores than the control groups (MD = 0.54, 95% CI −0.64 to 1.72, *P* = 0.37, Fig. 4e). A fixed-effects model was performed because of low heterogeneity (*I*^2^ = 0%, *P* = 0.55). Likewise, the KSS function scores were also similar between the two groups (MD = −2.47, 95% CI −11.62 to 6.68, *P* = 0.60). A random-effect model was used due to significant heterogeneity (*I*^2^ = 56%, *P* = 0.13; Fig. 4f).

### Complications

**Figure 5:**
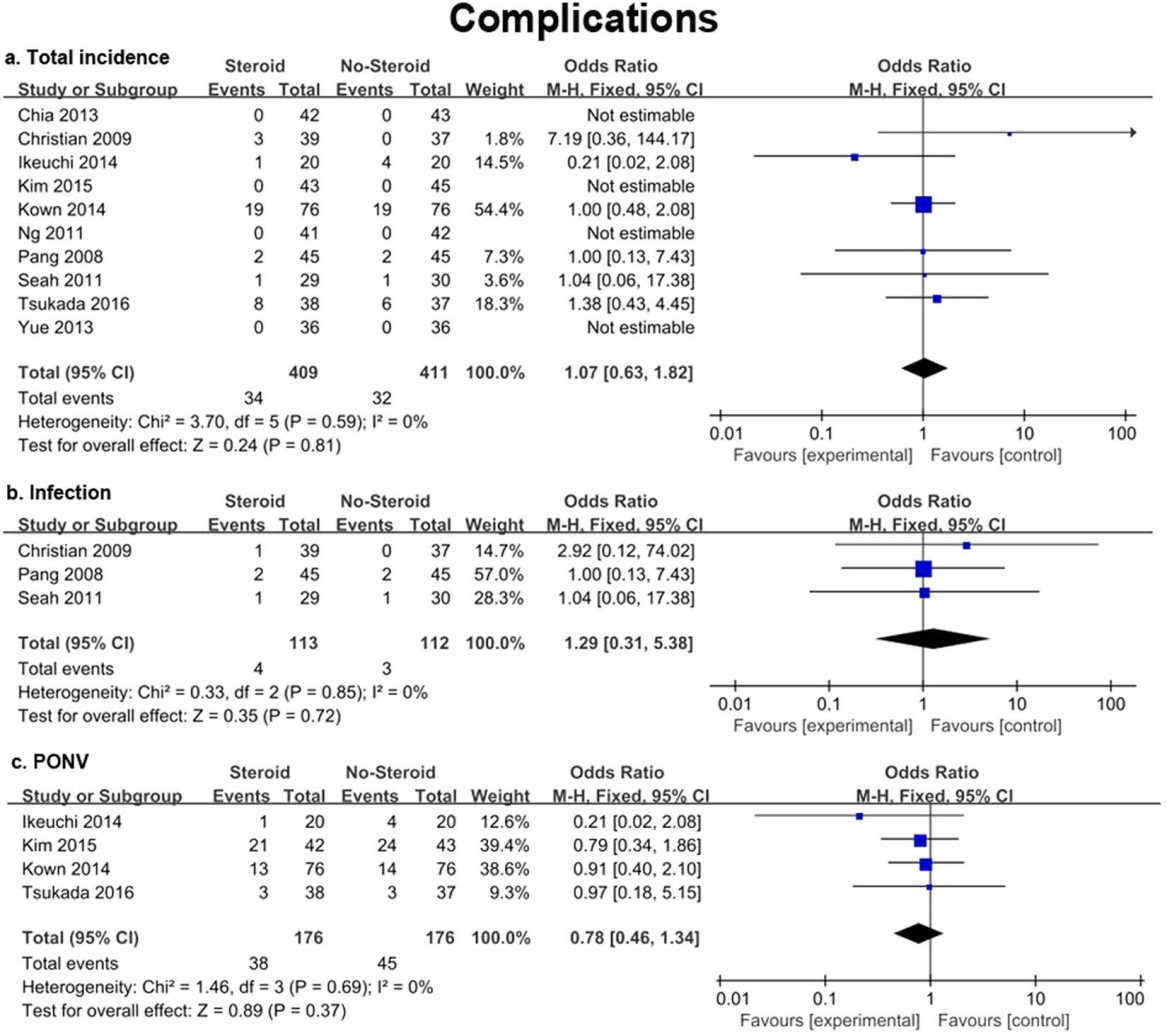
Forest plot diagram showing total incidence of complications (**a**), incidence of infection (**b**) and PONV (**c**) after knee joint arthroplasty.

All ten included studies reported the incidence of various complications such as PONV, infection, and wound oozing. There was no significant difference in total incidence of complications between the steroid and control groups (OR = −1.07, 95% CI 0.63 to 1.82, *P* = 0.81; Fig. 5a).

There were only two types of complications mentioned in at least two papers. Three studies^14,21,39^ involving 225 patients reported the occurrence of postoperative infection, and the steroid groups did not have an increased number of patients with infection (OR = 1.29, 95% CI 0.31 to 5.38, *P* = 0.72; Fig. 5b). Four trials^33–35,37^ involving 352 patients were included that assessed the occurrence of PONV. According to the outcomes, the incidence of PONV in the two groups was also similar (OR = 0.78, 95% CI 0.46 to 1.34, *P* = 0.37; Fig. 5c). The fixed-effects models were applied in these 4 cases due to the low heterogeneity (*I*^2^ = 0%). All the results of this meta-analysis are presented in Table 3.

**Table 3:** Results of Meta-Analysis.

Outcomes	Studies	No. of Patients	OR or MD (95% CI)	Heterogeneity (I^2^), %	*P*
**VAS**					
POD1	3	190	−1.52 (−2.94, −0.10)	82	0.04
POD3	2	110	−0.83 (−2.30, 0.64)	76	0.27
POW2	2	124	−0.42 (−1.19, 0.35)	14	0.29
**ROM**					
POD1	4	324	11.57 (9.85, 13.30)	47	<0.00001
POD2	3	248	9.03 (6.67, 11.38)	0	<0.00001
POD3	2	165	5.73 (0.85, 10.60)	44	0.02
POD4	2	165	5.53 (0.68, 10.38)	40	0.03
POD5	2	165	5.90 (0.87, 10.93)	0	0.02
POW1	2	227	1.46 (−1.21, 4.13)	0	0.28
POW2	2	160	−1.27 (−5.83, 3.29)	0	0.59
POW4	POW1	POW1	POW1	POW1	POW1
POW6	3	313	0.91 (−1.65, 3.47)	14	0.49
POM3	4	326	0.19 (−6.79, 7.17)	93	0.96
POM6	3	248	1.84 (−1.41, 5.08)	81	0.27
Morphine consumption	3	206	−7.94 (−14.35, −1.53)	73	0.02
CRP	2	123	−4.82 (−7.41, −2.23)	84	0.0003
SLR	3	282	−0.65 (−0.86, −0.44)	43	<0.00001
LOS	2	159	−0/98 (−1.25, −0.71)	0	<0.00001
**KSS (POM6)**					
Knee scores	2	228	0.54 (−0.64, 1.72)	0	0.37
Function scores	2	228	−2.47 (−11.62, 6.68)	56	0.60
Complications	10	820	1.07 (0.63, 1.82)	0	0.81
Infection	3	225	1.29 (0.31, 5.38)	0	0.72
PONV	4	352	0.78 (0.46, 1.34)	0	0.37

## Discussion

This meta-analysis assessed the efficacy and safety of the addition of steroids to MCPI for pain management and physical dysfunction of TKA and UKA patients. The current evidence shows that periarticular steroids injection is certainly effective in pain relief and improved ROM during the early stage after surgery, and is able to reduce morphine consumption, decrease CRP level, achieve earlier SLR, and shorten LOS, but the analgesic effect and functional outcomes were not significant compared to placebo groups during the late postoperative period. The most encouraging result is that the periarticular steroids injection did not increase the risk of postoperative infection, PONV, or other complications. Therefore, we conclude that steroids are more likely to play a positive role during the early postoperative period.

The forest plots indicated that the addition of steroids could further improve the analgesic efficacy of MCPI during the hyper-acute phase as we observed VAS score significantly decreased on the POD1 in the steroids group. The suppression of the inflammatory response by steroids application can be explained by the signaling pathways that suppress the cytoactivity of immune cells by inhibiting the production of phospholipase A2 (PLA2) and hence decreasing the production of inflammatory cytokines and chemokines^41^. Previous studies had detected lowered interleukin (IL)-6 and serum CRP level in cases treated with systemic and local steroids^37,40^. Our results also found decreased serum CRP level on the third day after surgery, proving the anti-inflammatory effects of steroids. Consistently, with the relief of postoperative pain, morphine consumption during the hospitalization was significantly decreased, and the time required to perform a SLR and LOS were also shortened in the steroids group. However, analgesic effect in the steroids group was not significant at POD3 and POW2, which indicated that MCPI with steroids could not provide a sustained analgesic effect. Steroids might play a pivotal role, but the effect of local anesthetics would disappear gradually over time.

In this meta-analysis, significant improvement in ROM was observed during the early postoperative period in those patients who received steroid injections during surgery, but the effects subsequently vanished. We inferred that the analgesic effect is sufficient to suppress the postoperative pain caused by surgical trauma, which is consistent with achievement of early SLR and shorter LOS as verified by our results. The difference in ROM between steroids and the control groups was insignificant at the POW1, suggesting that the addition of steroidsto MCPI was unable to increase the ROM, which might be explained by its inability to eliminate postoperative fibrosis and scarring within their respective time frame. Likewise, we observed no improvement of KSS knee or functional scores, which is a comprehensive system rating both the knee prosthesis function and patients’ functional abilities after TKA, at POW6. Three included RCTs reported the KSS scores. No group differences were noted in studies conducted by Christian *et al*. and Kown *et al*.^21,35^. Yue *et al*. observed significant improvement in postoperative KSSscores in the steroids group as compared with that in control groupat POM1 and POM3. However, at POM6, the KSS scores showed no significant difference between these two groups^36^. All these results indicate that long term effects of steroids application on functional outcomes are still uncertain.

For safety concerns, many surgeons remain leery of using steroids that may increase the risk of catastrophic complications such as infection and patellar tendon rupture^42,43^. A series of complications, including postoperative infection, wound oozing, PONV, pruritis, and transient peroneal nerve palsy were mentioned in the included studies. Our results indicate there were no difference in the total incidence of complications between the two groups. Steroids addition to MCPI would not increase the incidence of postoperative infection and PONV. Furthermore, there were no differences in the incidences of complicationsbetweenthe steroids and placebo groups. No case of tendon rupture was reported in the included RCTs.

Limitations of the present meta-analysis should also be acknowledged. Firstly, the sample size is relatively small and some of the indexes were objectively judged which may reduce the reliability of the study. The second limitation is the lack of consensus on the type and doses of the drugs applied in MCPI which also undermine the consolidation of the analysis. Third, some data were missing or could not be extracted. Some of the results appeared heterogeneous, and couldn’t be eliminated by sensitivity or subgroup analyses. We included these high-quality studies and applied random-effect models for meta-analysis, which may mildly influence the reliability of the results. Finally, the reported side effect is scarce and duration of follow-up is relatively short, which might lead to underestimation of complications and uncertainty of long-term efficacy^44^. Despite the above limitations, this is the first meta-analysis that included RCTs to evaluate the efficiency and safety of periarticular steroids addition to MCPI in TKA and UKA. Large well-designed RCTs are still needed to validate this research moving forward.

In conclusion, this meta-analysis of RCTs suggested that the administration of periarticular steroids injection with MCPI appears to effectively relieve postoperative pain, improve functional outcomes, reduce morphine consumption, and decrease inflammatory reaction without causing a higher incidence of complications in the early postoperative period.

## Methods

### Search strategy

This quantitative meta-analysis was in accordance with the Preferred Reporting Items for Systematic Reviews and Meta-analyses (PRISMA) guidelines^45^. Two researchers searched the relevant studies independently including MEDLINE/PubMed database, the Cochrane Central Register of Controlled Trials (CENTRAL), and EMBASE databases from inception to April, 2018 for relevant RCTs that compared postoperative periarticular injection of steroids with placebo in the knee arthroplasty. Search terms included “knee”, “arthroplasty”, “replacement”, “steroids”, “corticosteroids”, “cortisol”, “cortisone”, “dexamethasone”, “glucocorticoid”, “betamethasone”, “hydrocortisone”, “prednisone”, “prednisolone”, “ethylprednisolone”, “riamcinolone”, and “adrenal cortex hormone” (supplymentary files). Search terms were combined using the Boolean operators ‘AND’ or ‘OR’. No restrictions were imposed, and reference lists of retrieved articles and reviews were also searched. A third reviewer acted as a judge if there was any disagreement.

### Inclusion and exclusion criteria

Only RCTs comparing the clinical efficacy between postoperative periarticular or intraarticular injection of steroids with placebo among adults of any sex undergoing primary KA (including TKA, PKA, UKA, *et al*.) for any indication (osteoarthritis, rheumatoid arthritis, osteonecrosis or acute trauma) were included in our study. Studies that reported at least one outcome were included. The Primary outcomes being VAS and ROM. With secondary outcomes being morphine consumption, CRP, SLR, LOS, and KSS knee and function scores. Studies were excluded from present meta-analysis in view of incomplete data, letters, comments, editorials, case reports, conference abstracts, or review articles.

### Data extraction

The relevant data was extracted from included studies by using a standard data extraction. Two researchers used this form to collect the information from studies independently. The main characteristics included the following items: first author’s name, publication year, country, sample size, mean age, sex ratio, BMI, steroids intervention (dose and type), anesthetic techniques, perioperative analgesia intervention, and duration of follow-up. Other relevant data were extracted from included studies. Differences were resolved by consensus. If a study reported the outcomes of multiple doses of steroids for treatment, only data of the low dose group was extracted for analysis. The first main index was VAS score that has 11 pain levels (0 = no pain, 10 = extreme pain). All other types of VAS were converted to this 11 levels score according to ratio. The second index was ROM, composed of both flexion and extension angles of the knee. The flexion angle was used to represent ROM if flexion and extension angles cannot be combined. The third index contained the postoperative morphine consumption. The total amount of morphine consumption during hospitalization was accumulated for analysis. The other indexes included CRP, SLR, LOS, KSS knee and function scores, and complications. CRP is an acute-phase protein synthesized by the liver which reflects the postoperative inflammation^46^. All the data on CRP were converted to the unit of mg/dl. SLR is a test-conducted post knee surgery to gauge how high a patient is able to elevate his/her leg off of an exam table, reflecting pain control as well as muscle strength recovery^26^. KSS, composed of knee score and function score, is considered as an objective scoring system to rate the knee and patient’s functional abilities such as walking and stair climbing before and after TKA^47^. The total incidence of complications after surgery was calculated. If any special type of complication was mentioned more than twice, its incidence was pooled for subgroup analysis^48^. For missing or incomplete data, we extracted information from diagrams or contacted the corresponding authors to ensure that the information was integrated. Otherwise, we estimated the data wherever possible using the recommendations in the Cochrane Handbook^49^. Formats that were not suitable included studies reporting means with interquartile ranges, suggesting the data were non-normally distributed making conversion to means and SDs controversial^49^. Furthermore, data only reporting means without SDs, standard errors (SEs) or confidence intervals (CIs) could not be extracted considering that one of these is required with the mean for meta-analysis.

### Quality assessment

The risk of bias of the included studies was assessed using the Cochrane Handbook for Systematic Reviews of Interventions version 5.1.0^49^, based on the following items: random sequence generation, allocation concealment, blinding of participants and personnel, blinding of outcome assessment, incomplete outcome data, and selective reporting. The assessment items were either categorized as low risk of bias, high risk of bias, or unclear risk of bias. Discrepancies between assessments were resolved by discussion with a third reviewer when necessary.

### Statistical analysis

For continuous data, like VAS scores, ROM and so on, we calculated mean differences (MDs) and 95% CIs if outcomes were measured in the same way between studies, or we used the standardized mean differences (SMDs). Continuous data reported as means and ranges were transformed into means and SDs using Hozo’s formula^50^. Dichotomous data such as incidence of complications were expressed as odds ratios (ORs) with 95% CIs indicating the effect on intervention. Subgroup analysis was performed for different time points. The Q and chi-squared test were used to assess statistical heterogeneity with the value of *P* and *I*^2^. If heterogeneity was low (*P* > 0.1, *I*^2^ < 50%), a fixed-effects model was used. If heterogeneity was significant (*P* < 0.1, *I*^2^ > 50%), a random-effects model was used. When possible, sensitivity and subgroup analyses were conducted to find the source of the heterogeneity. Publication bias was visually examined by funnel plots. We used Review Manager 5.2 software (Rev Man 5.2, The Cochrane Collaboration, Oxford, UK) and STATA, version 12.0 (Stata Corp LP, College Station, TX) to perform statistical analyses. *P* < 0.05 was considered statistically significant.

## Supplementary information

Search Strategy
