# Supplementary material for: The efficiency and safety of steroid addition to multimodal cocktail periarticular injection in knee joint arthroplasty: a meta-analysis of randomized controlled trials
Source: Sci Rep. 2019 May 7;9:7031. doi: 10.1038/s41598-019-43540-9 (PMC6505038; doi:10.1038/s41598-019-43540-9)
Supplement: Supplementary file 1 — Search Strategy [file 41598_2019_43540_MOESM1_ESM.docx]

**The efficiency and safety of steroid addition to multimodal cocktail periarticular injection in knee joint arthroplasty: a meta-analysis of randomized controlled trials**

Zhenhan Deng, Yusheng Li, Garrett R. Storm,RonakNaveenchandra Kotian, Xuying Sun,Guanghua Lei, Shanshan Gao, Wei Lu

Pubmed:

(((((arthroplasty[Title/Abstract]) OR replacement[Text Word])) AND knee[Title/Abstract])) AND (((((((((((((steroid[Title/Abstract]) OR corticosteroid[Title/Abstract]) OR cortisol[Title/Abstract]) OR cortisone[Title/Abstract]) OR glucocorticoid[Title/Abstract]) OR betamethasone[Title/Abstract]) OR dexamethasone[Title/Abstract]) OR hydrocortisone[Title/Abstract]) OR prednisone[Title/Abstract]) OR prednisolone[Title/Abstract]) OR methylprednisolone[Title/Abstract]) OR triamcinolone[Title/Abstract]) OR adrenal cortex hormone[Title/Abstract])

296

Cochrane Library

#1 arthroplasty or replacement 26543

#2 knee 18454

#3 #1 and #2 5491

#4 steroid or corticosteroid or cortisol or cortisone or glucocorticoid or betamethasone or dexamethasone or hydrocortisone or prednisone or prednisolone or methylprednisolone or triamcinolone or adrenal cortex hormone 50397

#5 #3 and #4 230

Trials：127

Embase

#1. 'arthroplasty':ti,ab,kw OR 'replacement':ti,ab,kw 344,119

#2. 'knee':ti,ab,kw 156,713

#3. #1 AND #2 36,350

#4. 'steroid':ti,ab,kw OR 'corticosteroid':ti,ab,kw 446,814

OR 'cortisol':ti,ab,kw OR 'cortisone':ti,ab,kw OR

'glucocorticoid':ti,ab,kw OR

'betamethasone':ti,ab,kw OR

'dexamethasone':ti,ab,kw OR

'hydrocortisone':ti,ab,kw OR

'prednisone':ti,ab,kw OR 'prednisolone':ti,ab,kw

OR 'methylprednisolone':ti,ab,kw OR

'triamcinolone':ti,ab,kw OR 'adrenal cortex

hormone':ti,ab,kw

#5. #3 AND #4 495
